# Editorial: Biomarkers and aging: new insights for precision medicine

**DOI:** 10.3389/fragi.2026.1987600

**Published:** 2026-09-21

**Authors:** Oluwafemi Gabriel Oluwole, Paulo Adriano Schwingel

**Affiliations:** 1 Computational Pharmacology and Genomics Research Group, Department of Pharmacology and Therapeutics, Olabisi Onabanjo University, Sagamu, Ogun State, Nigeria; 2 Institute of Infectious Diseases and Molecular Medicine, University of Cape Town, Cape Town, South Africa; 3 Human Performance Research Laboratory (LAPEDH), University of Pernambuco (UPE), Petrolina, Brazil; 4 AI-assisted Diagnostics Research Group (AIDRG), University of Pernambuco (UPE), Petrolina, Brazil

**Keywords:** aging, algorithm, biomarker, genomics, immune response

## Introduction

Aging is a complex and heterogeneous biological process marked by progressive molecular, cellular and physiological change and increasing susceptibility to chronic disease. Chronological age captures little of this variability: individuals of the same age can differ substantially in physiological resilience, disease risk and functional capacity. Understanding these differences is central to extending healthy lifespan and to individualizing prevention and treatment. This Research Topic, *Biomarkers and Aging: New Insights for Precision Medicine*, brings together emerging biomarkers that may improve prediction, diagnosis, management and prognosis of age-related conditions, with attention to molecular mechanisms—genetic and epigenetic regulation, inflammation, immune and stress responses, DNA repair—and to population diversity in precision medicine.

The four contributions gathered here trace a coherent progression, from the conceptualization of biological age to its molecular measurement, computational interpretation and clinical relevance. Together, they show that biological aging is a dynamic, multidimensional phenomenon shaped by molecular regulation, cellular function, physiological systems and individual exposures, not a process any single biomarker or score can capture.

## From molecular markers to computational measures of aging

DNA methylation-based measures of biological age have become one of the most influential tools in the field. Ziesel et al. provide a scoping review of 435 studies, 167 of which used methylation-based approaches, tracing the progression from age-associated CpG patterns to increasingly sophisticated epigenetic clocks. A companion review by the same group extends this analysis to 309 non-methylation-based biomarkers. The diversity of markers and metrics reinforces that biological aging is unlikely to be captured by a single clock. Read together, these two reviews shift the field’s focus: from searching for the single best biomarker toward identifying combinations that capture the dimensions of aging relevant to a given individual and clinical outcome. A person may show accelerated epigenetic aging alongside preserved cardiovascular function, or metabolic and immune aging despite a modest global biological-age estimate—a single number can inform while concealing real heterogeneity.

## From biological age to clinical relevance

Chen et al. bridge biological-age assessment and clinical outcomes using data from 349,456 UK Biobank participants. Over roughly 14 years of follow-up, phenotypic age acceleration partially mediated the association between impaired lung function and both mortality and cardiovascular disease, with risk highest in participants showing both. Biological age may capture accumulated vulnerability that chronological age or a single organ measurement misses. Though this reflects biological aging as an integrative indicator of interacting processes, not direct causation, its clinical value will likely be to complement established risk factors, not replace them.

## Proteomics and the physiological dimension

Proteomics adds a functional dimension to this picture. Luo et al. review proteomic biomarkers across the immune, metabolic, endocrine, cardiovascular, musculoskeletal and nervous systems, together with proteomic aging clocks linked to frailty, multimorbidity, organ-specific biological age and mortality. Proteins are functional effectors rather than regulatory markers, so they complement epigenetic information with insight into downstream biological activity. That adds to a multidimensional aging profile rather than a single biological-age estimate. Related physiological signals point the same way: Oluwole and Schwingel found that reduced heart-rate-variability indices (a marker of autonomic and parasympathetic modulation) were associated with cognitive decline in community-dwelling older adults. Functional, system-specific biomarkers like this extend molecular measures of biological age; they do not replace them.

## From biomarkers to mechanisms and therapeutic discovery

Moving from biomarker measurement to precision medicine also means linking molecular information to candidate interventions. Oluwole and Schwingel combined transcriptomic analysis of human Achilles tendon tissue with sequence analysis, molecular docking and protein–protein interaction analysis to investigate a small peptide derived from methyl jasmonate esterase and its interaction with human complement factor H. The approach shows how computational methods can move from biomarker identification to mechanistic, testable hypotheses. A related study, by Oluwole and Schwingel, applied network pharmacology and molecular docking to Sorghum bicolor 3-deoxyanthocyanidins, identifying inflammation- and redox-related pathways (NF-κB, Keap1–Nrf2, PPAR-γ) with experimental support for modulating inflammaging-related processes. Neither example shows an established anti-aging therapy. Both illustrate an emerging pathway in which biomarkers and mechanisms help prioritize hypotheses for further, experimentally validated investigation. Figure 1 illustrates how these biomarker, mechanistic and computational strands can be integrated into a single translational framework for precision-medicine approaches to aging.

**FIGURE 1:**
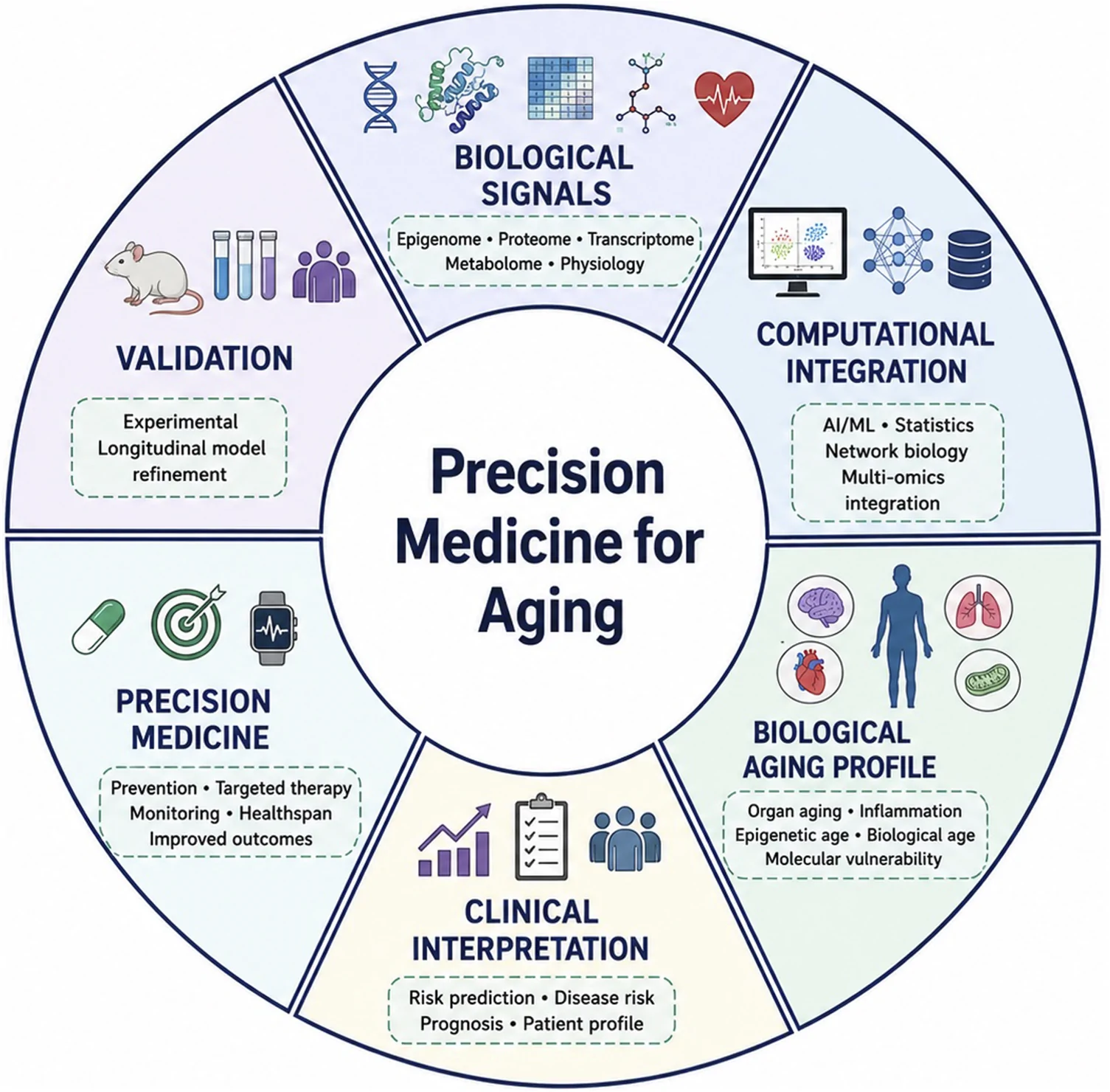
From biomarker discovery to precision medicine for biological aging. Epigenetic, proteomic, physiological and mechanistic biomarkers of aging can be integrated through computational and statistical approaches into individual biological-aging profiles, supporting risk prediction, clinical validation and targeted intervention, with longitudinal evidence iteratively refining biomarker selection and interpretation.

## Population diversity and the path to intervention

Whether biomarkers and computational clocks developed in specific populations generalize across genetic backgrounds, environments and socioeconomic circumstances remains an open, consequential question: precision medicine cannot be precise if its underlying biomarkers under-represent the populations in which they will be applied. Future work should prioritize diverse cohorts and rigorous external validation across geographic and ancestral contexts. A parallel question concerns actionability. A biomarker that predicts disease is scientifically valuable, but its clinical worth depends on whether it identifies modifiable processes and supports interventions that improve outcomes. That will require longitudinal studies tracking whether biological-age measures respond to lifestyle or pharmacological intervention, and whether those changes translate into meaningful gains in healthspan.

## Conclusion

Together, these contributions illustrate a field moving from the search for individual biomarkers toward an integrated understanding of biological aging as dynamic and multidimensional. Epigenetic, proteomic, physiological and mechanistic approaches each contribute a piece of this picture; the next stage will depend on integrating them, through multi-omics measurement, computational modelling and diverse longitudinal cohorts, while keeping models reproducible, interpretable and clinically validated. Precision medicine for biological aging will ultimately depend on moving beyond measuring biological age toward understanding biological vulnerability, and on using that understanding to preserve healthspan. Figure 1 summarizes this translational pathway, from multidimensional biomarker discovery to clinical application.

